# Te/Fe_3_GaTe_2_ 1D-2D Ferroelectric Heterojunction Transistors Enabling Ultrafast Multi-State Switching for Workpiece Surface Defect Inspection

**DOI:** 10.3390/nano16150935

**Published:** 2026-07-29

**Authors:** Shiqiang Wang, Zewei He, Tianyun Wang, Ziyu Gao, Lin Wang, Jinlei Zhang, Yucheng Jiang

**Affiliations:** 1 School of Materials Science and Engineering, Shanghai University, Shanghai 200444, China; 2 School of Physical Science and Technology, Suzhou University of Science and Technology, Suzhou 215009, China

**Keywords:** Te nanowire, van der Waals heterojunction, ferroelectric semiconductor field-effect transistor, neuromorphic computing, nonvolatile memory

## Abstract

Ferroelectric field-effect transistors, which rely on ferroelectric polarization reversal to modulate the channel resistance, hold great promise for nonvolatile memory and neuromorphic computing. The polarization switching dynamics are critical for achieving high-speed, high-bit-density neuromorphic hardware. Here, we report a 1D-2D asymmetric heterojunction composed of a single-element tellurium (Te) nanowire and a magnetic metal, Fe_3_GaTe_2_. Piezoresponse force microscopy reveals reversible polarization switching at room temperature. Utilizing this ferroelectric heterojunction, we construct ferroelectric semiconductor field-effect transistors that exhibit tunable resistance states exceeding 7 bits, featuring an on/off ratio of 10^3^, a retention time exceeding 10^3^ s, and ultrafast switching down to 20 ns. Moreover, the transistor enables accurate recognition of six kinds of micro-defects with an accuracy of 97.1% on the workpiece surface by convolutional neural network. This work establishes the intrinsic relationship between ferroelectric polarization and resistance modulation, providing a device platform for next-generation multilevel storage and ultrafast neuromorphic computing networks.

## 1. Introduction

Ferroelectrics sustain a depolarization field under remanent polarization in the absence of an external electric field [1,2,3]. The direction of this depolarization field can be reversed by an external electric field, providing a physical basis for nonvolatile multilevel resistance states in ferroelectric materials [4,5,6,7]. When a ferroelectric is employed as the gate insulator, the channel conductance can be switched by the polarization state, enabling the realization of ferroelectric field-effect transistors (FeFETs) [8,9,10,11]. Driven by polarization reversal, these FeFETs represent a promising memory technology because of their fast switching speed, continuously tunable (analog) memory states, and high data density suitable for high-density integration [12,13,14,15,16]. To date, various hardware implementations have been intensively explored to emulate synaptic functions, including metal-oxide-based memristive systems [17], phase-change memory (PCM) arrays [18], halide perovskites [19], and self-assembled nanowire networks [20], each offering unique advantages in speed, scaling, or bio-compatibility. Among these, ferroelectric-gate architectures remain highly attractive. However, conventional FeFETs [21,22,23,24,25,26,27] suffer from severe charge screening effects at the ferroelectric/semiconductor interface, which often induce unpredictable threshold voltage variations and thus impede stable multilevel resistance state programming [28,29,30].

An alternative device architecture, the ferroelectric semiconductor field-effect transistor (FeS-FET), employs a ferroelectric semiconductor as the channel material [31,32,33]. In this configuration, the out-of-plane depolarization field modulates the in-plane transport properties, thereby enabling nonvolatile resistive switching directly within the channel [34,35]. Consequently, FeS-FETs can potentially eliminate the charge screening effects and leakage currents that plague conventional FeFETs at the ferroelectric insulator/semiconductor interface [36,37]. Recently, FeS-FETs based on In_2_Se_3_ layers have been reported, demonstrating a high on/off ratio and low power consumption, establishing a promising direction for further research on FeS-FETs [38,39,40]. However, despite the intrinsic advantages of ferroelectric semiconductors—namely the high speed of polarization reversal and the capability for partial domain reversal—the switching speed and the number of achievable nonvolatile resistance states in FeS-FETs remain largely unexplored [41,42,43,44,45].

Here, we construct a FeS-FET by integrating a single-element ferroelectric Te nanowire with a 2D metallic ferromagnet Fe_3_GaTe_2_ to form a 1D-2D lateral heterojunction, where the heterojunction serves as the ferroelectric and channel layer. When electrical pulses are applied through the Si/SiO_2_ to modulate polarization reversal, the FeS-FET exhibits long-term retention exceeding 10^3^ s, an ultrafast switching speed down to 20 ns, and more than 128 nonvolatile resistance states. These performance characteristics are primarily attributed to the fast reversal of ferroelectric polarization and the partial switching of ferroelectric domains. The simulation, based on a convolutional neural network (CNN), shows that the device enables high-accuracy identification of micro-defects on workpiece surfaces.

## 2. Results and Discussion

### 2.1. Structural Characterization of 1D-2D Te/Fe_3_GaTe_2_ Heterojunctions

**Figure 1:**
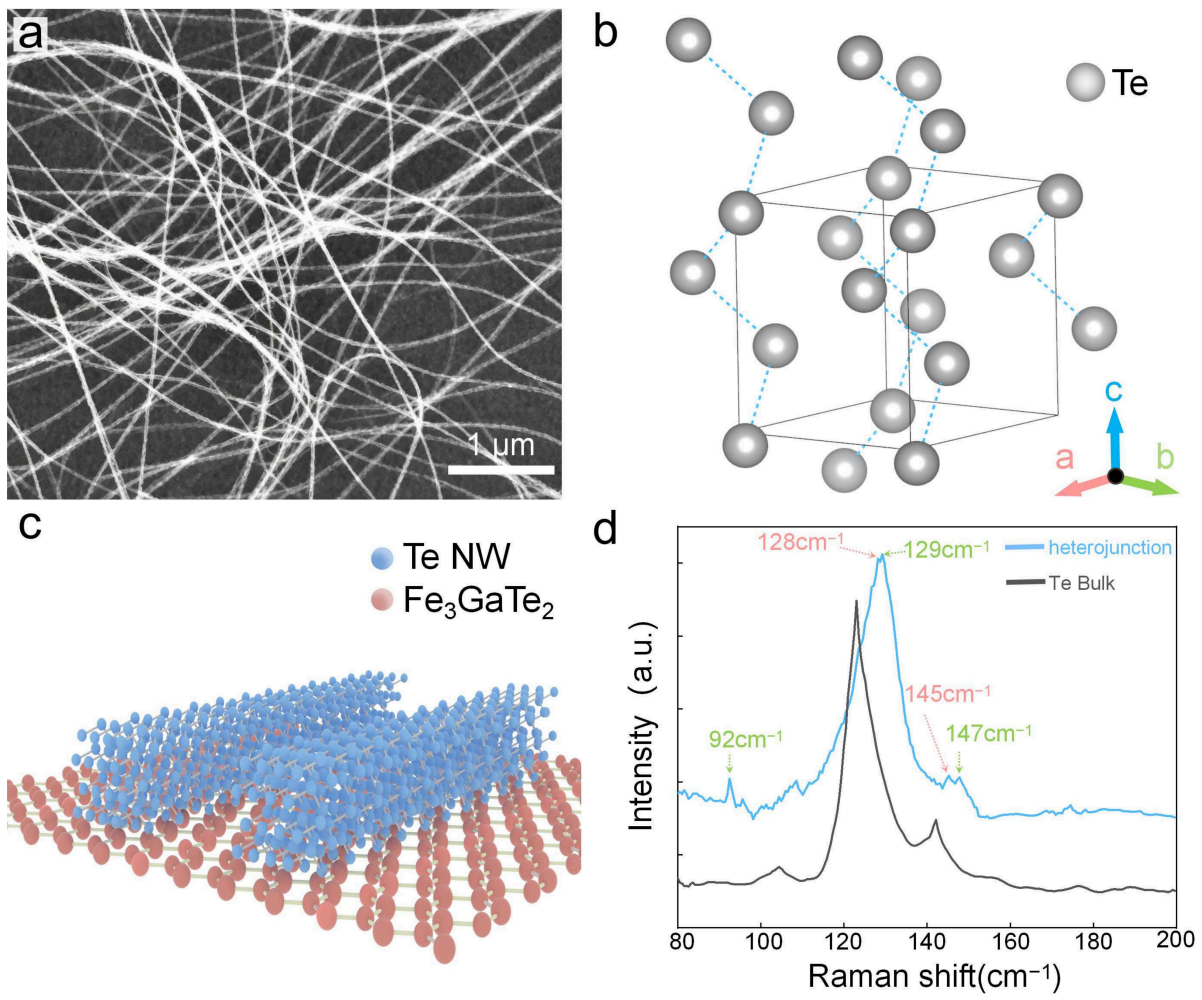
Morphology and atomic arrangement of the Te nanowire. (**a**) SEM morphology images of Te nanowires on a SiO_2_/Si wafer. (**b**) The atomic lattice models of Te nanowires. (**c**) Illustration image of a 1D-2D Te/Fe_3_GaTe_2_ heterojunction. (**d**) Raman spectra of Te and Fe_3_GaTe_2_ in the 1D-2D Te/Fe_3_GaTe_2_ heterojunction and bulk Te.

Quasi-1D single-crystal Te nanowires were synthesized via a bottom-up hydrothermal method. Scanning electron microscopy (SEM) reveals that the as-grown Te nanowires have lengths exceeding tens of micrometers and widths below 100 nm (Figure 1a). The as-prepared Te nanowire exhibits a trigonal crystalline phase (space group P3_1_21) and a characteristic chain-like structure along the c-axis (Figure 1b), indicating high crystallinity. Mechanically exfoliated 2D Fe_3_GaTe_2_ flakes were precisely positioned onto one end of a single Te nanowire using a dry transfer technique. Thus, a cross-dimensional 1D-2D lateral Te/Fe_3_GaTe_2_ heterojunction can be constructed (Figure 1c). Raman spectroscopy (Figure 1d) reveals distinct characteristic peaks of Te at 92 cm^−1^, 129 cm^−1^, and 147 cm^−1^ (green), and of Fe_3_GaTe_2_ (red) at 128 cm^−1^ and 145 cm^−1^, confirming the successful preparation of the Te/Fe_3_GaTe_2_ heterojunction. Notably, compared with bulk Te crystals (black), the Te nanowire in the heterojunction exhibits not only broadening of the E_1_-TO phonon mode but also the emergence of the E_1_-LO vibrational mode, indicating a reduction in crystal symmetry.

### 2.2. Ferroelectric Characteristics of the 1D-2D Te/Fe_3_GaTe_2_ Heterojunction

**Figure 2:**
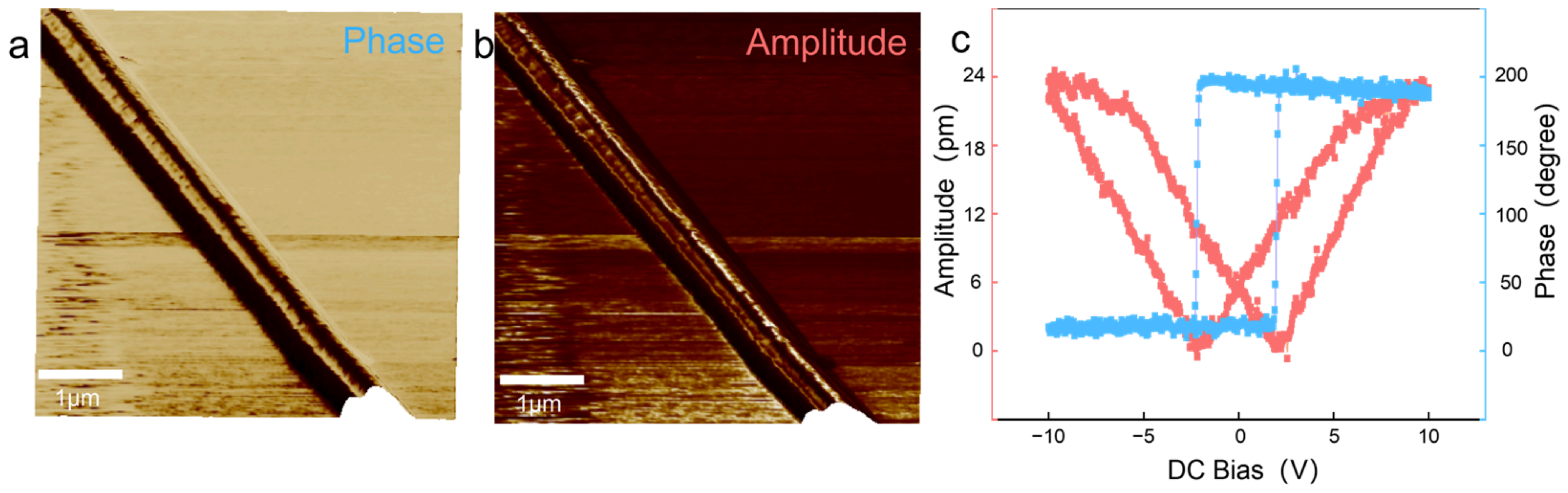
Ferroelectric hysteresis and domain of the 1D-2D Te/Fe_3_GaTe_2_ heterostructure. (**a**,**b**) Phase (**a**) and amplitude (**b**) mapping of domain measured by vertical PFM in the Te/Fe_3_GaTe_2_ heterostructure. (**c**) Phase-voltage (blue) and amplitude-voltage (red) hysteresis loops measured by vertical OFF-field PFM in Te/Fe_3_GaTe_2_ heterostructure.

To probe the ferroelectricity of the Te nanowire within the lateral Te/Fe_3_GaTe_2_ heterojunction, vertical piezoresponse force microscopy (PFM) measurements were strictly on both the bare Te nanowire regions adjacent to the junction and the heterojunction overlap region. To exclude artifacts arising from charge trapping/detrapping processes, an off-field mode PFM measurement with the tip bias removed was employed to characterize the intrinsic ferroelectric response and domain structure. The phase (Figure 2a) and amplitude (Figure 2b) mappings, overlaid on the topography, reveal a belt-like domain distribution and demonstrate excellent piezoelectricity with an effective piezoelectric coefficient d_33_ of approximately 2.5 pm V^−1^. Off-field vertical PFM measurements conducted on this bare Te nanowire region near the junction further yield a characteristic phase hysteresis loop (blue) and amplitude butterfly loop (red), as shown in Figure 2c. Both loops exhibit a coercive voltage of approximately ±2.5 V, consistent with the switching voltages derived from the phase and amplitude responses. Furthermore, PFM measurements were also conducted on the Te nanowire in the heterojunction overlap region. Similar PFM hysteresis loops can also be observed in Figure S1. These ferroelectric characteristics confirm that the lateral Te/Fe_3_GaTe_2_ heterojunction retains robust ferroelectricity after integration. This establishes the essential physical basis for subsequently modulating the channel conductance via the polarization field to achieve nonvolatile resistive switching.

### 2.3. Multiple Nonvolatile Resistive Switching Caused by Domain Reversal in FeS-FET

The Te/Fe_3_GaTe_2_ ferroelectric heterojunction (~36 nm-thick Fe_3_GaTe_2_ and ~22 nm-high Te nanowire) functions as a lateral channel device featuring a localized vertical cross-dimensional overlap interface. The vertical remanent polarization of the Te nanowire directly modulates the in-plane transport properties of the carrier in the heterojunction. Physically, current flows laterally from one contact electrode, across the 1D-2D interface, and through the Te nanowire channel. Because Te is an intrinsic p-type semiconductor, the vertical polarization field of the Te nanowire tunes the carrier (hole) accumulation/depletion within the junction, effectively shifting the local Fermi level and modulating the barrier height.

**Figure 3:**
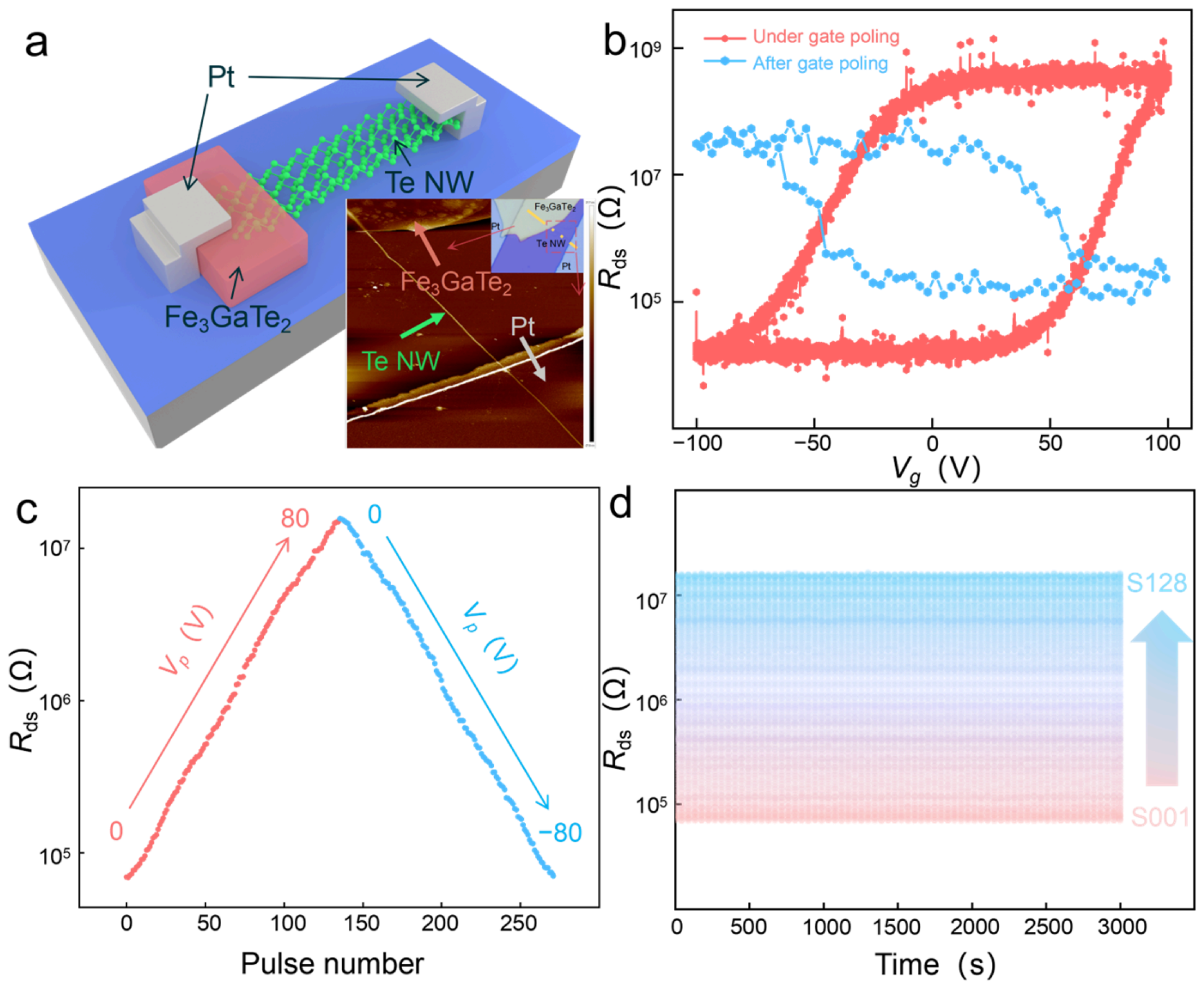
Switching characteristics exhibiting nonvolatile memory of a Te/Fe_3_GaTe_2_ heterostructure. (**a**) Schematic illustration of the memory device. (**b**) R_ds_-V_g_ characteristics for the Te/Fe_3_GaTe_2_ showing the nonvolatile memory after electric pulse. (**c**) Nonvolatile switching of R_ds_ measured after applying multiple V_p_ square pulses with the pulse width of 1 μs, exhibiting multilevel resistive states above 128. (**d**) Resistance retentions for 128 distinct resistive states for up to 3000 s after applying V_p_ with a pulse duration of 1 μs.

As a result, the FeS-FET exhibits R_ds_-V_g_ behavior under a continuous DC gate sweep, showing a typical transfer curve (red line) in the field-effect transistor in Figure 3b. The hysteresis loop is counterclockwise, with an ON/OFF ratio reaching 10^3^, confirming P-type carrier in the Te/Fe_3_GaTe_2_ heterojunction. In contrast, when a discrete gate pulse of amplitude V_p_ (1 μs width) is applied and then completely removed, the non-volatile remanent resistance state can be read out. This post-pulse state is plotted as the blue curve in Figure 3b. Under these conditions, the R_ds_-V_p_ hysteresis immediately switches to a clockwise direction, exhibiting nonvolatile resistive switching with an ON/OFF ratio of up to 10^2^ caused by the opposite remanent electric field. To further investigate the continuously tunable states, consecutive electrical pulses (1 μs width) with gradually increasing amplitudes were applied to the gate. The FeS-FET shows 128 distinct resistance states (Figure 3c), the resistance values of which decrease with gradually decreasing amplitudes, mimicking synaptic behavior in neurons. Subsequently, retention measurements performed on all 128 states reveal no significant degradation over 3000 s (Figure 3d), demonstrating highly stable long-term retention. To verify the statistical distinctness of these states, we extracted the mean values and standard deviations (σ) of the states during the 3000 s retention test, which are detailed in Figure S2. The calculated separation margins with error bars (±3σ) show zero statistical overlap between adjacent levels, proving the high reproducibility and low read noise of our device. Both HRS and LRS show long-term retention above 10^4^ s in Figure S3. Interface trapping/detrapping typically exhibits unstable retention spanning from several milliseconds to hundreds of seconds, confirming the origination of ferroelectric reversal.

These continuously tunable nonvolatile resistance states are attributed to the partial reversal of ferroelectric domains under successive pulse voltages. Combined with the miniature size of the ferroelectric domains, the device achieves multi-bit storage states. Furthermore, the ultrahigh information storage density is derived using the following formula:

$$Density\;\left(\frac{Tbit}{{in}^{2}}\right)=\frac{{in}^{2}}{A_{domain}}\times 7\;bits$$

where *A*_domain_ is the average area of a single ferroelectric domain. This yields a calculated system-level information density of approximately 1.19 Tbit inch^−2^.

### 2.4. Ultrafast Resistive Switching (20 ns) in FeS-FET

Taking advantage of the ultrafast reversal of ferroelectric polarization, we characterized the resistive switching speed of the FeS-FET by applying electrical pulses with a width of 20 ns. Here, the 20 ns refers strictly to the applied programming pulse width that successfully triggers non-volatile polarization reversal. We employed a high-bandwidth pulse generator connected through RF coplanar probe stations with 50 Ω impedance matching. The calculated parasitic RC delay of our microscale device is below 1 ns, ensuring that the applied 20 ns pulse is fully loaded across the channel heterojunction.

**Figure 4:**
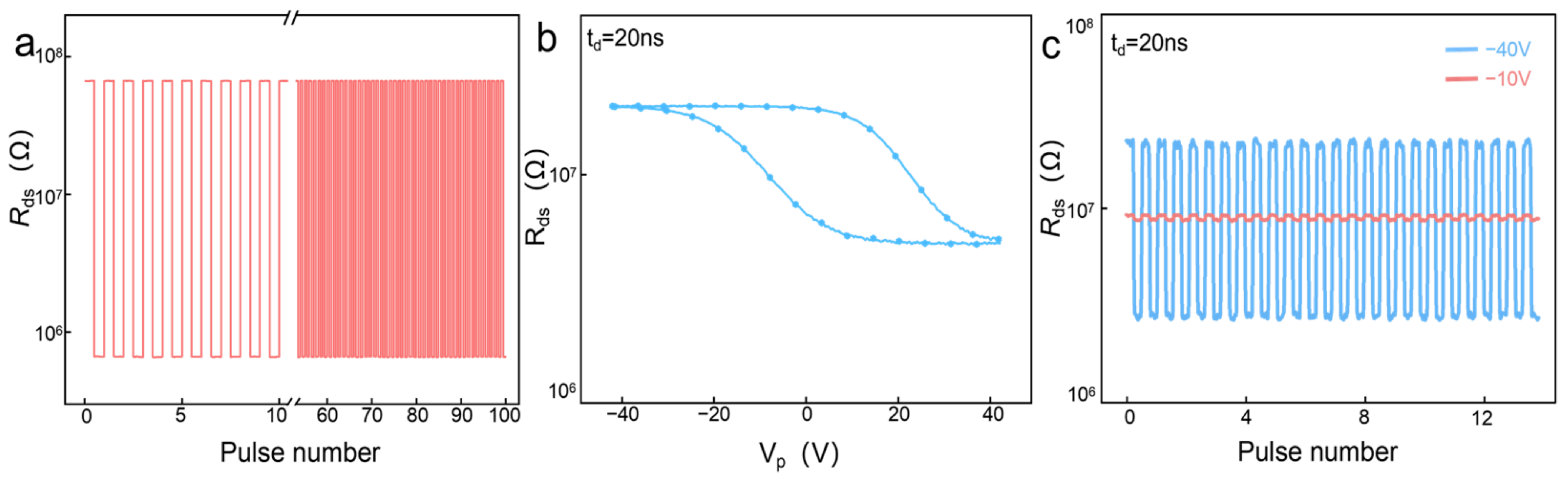
Fast nonvolatile switching of the Te/Fe_3_GaTe_2_ heterostructure. (**a**) Nonvolatile switching of R_ds_ measured after applying gate voltage pulses with durations of 20 ns. (**b**) R_ds_-V_p_ hysteresis loops showing the continuous-variable resistive states measured applying V_p_ pulse-width of 20 ns. (**c**) Switching dependence of R_ds_ on pulse amplitude after applying 20 ns pulses.

As shown in Figure 4a, the FeS-FET maintains stable switching characteristics under repeated 20 ns pulses. The experimental results reveal that the device exhibits nonvolatile bistable switching behavior with no obvious degradation after multiple cycles. Given that charge trapping/detrapping processes and electrochemical ionic migration typically occur on timescales exceeding 100 ns, the observed resistive switching can be reliably attributed to polarization reversal of the ferroelectric heterojunction. Therefore, the observed ultrafast nonvolatile bistable switching behavior is dominantly governed by the intrinsic, high-speed polarization reversal within the 1D-2D ferroelectric heterojunction.

Subsequently, when the pulse amplitude is varied while keeping the pulse width at 20 ns, the FeS-FET still yields a distinct clockwise R_ds_-V_p_ hysteresis loop (Figure 4b), demonstrating a wide memory window and confirming the feasibility of achieving continuously tunable resistance states even with 20 ns pulses. Furthermore, when the gate pulse amplitude is reduced to 10 V, no resistive switching is observed (Figure 4c), indicating that pulse voltages below the coercive voltage cannot induce switching. With its continuously tunable resistance states, ultrahigh data density, and ultrafast operation speed, the FeS-FET can be integrated with silicon circuits via three-dimensional heterogeneous integration to realize CMOS-compatible neuromorphic devices.

### 2.5. Identification of Micro-Defects on Workpiece Surfaces

**Figure 5:**
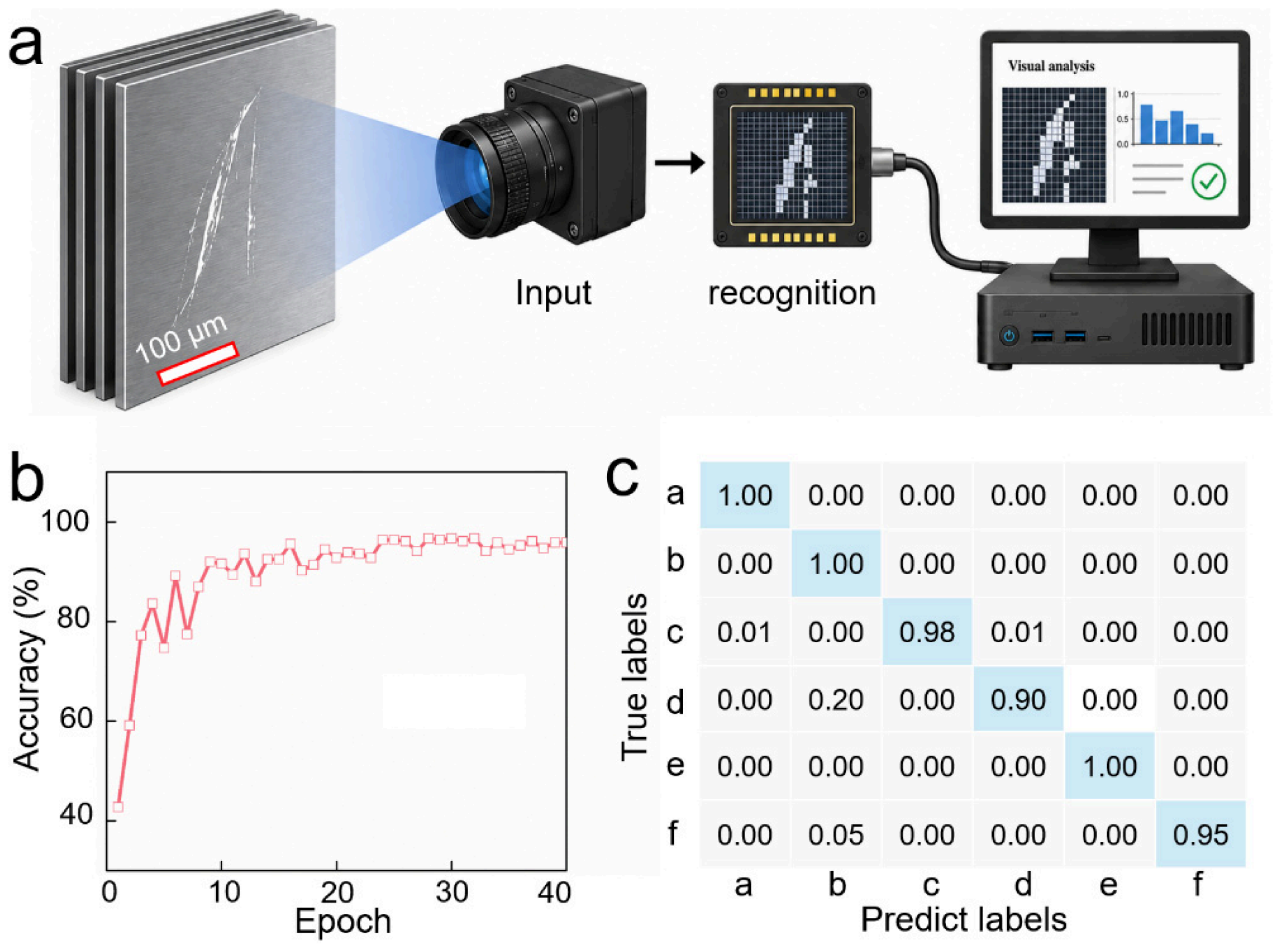
Simulations of micro-defects on workpiece surfaces with FeS-FET neuromorphic system. (**a**) Schematics of the neuromorphic system. (**b**) The defect recognition rate. (**c**) The confusion matrix shows high accuracy, with correct identifications of the six kinds of micro-defects.

To evaluate the application of the Te/Fe_3_GaTe_2_ FeS-FET for neuromorphic tasks, we performed comprehensive simulations of a CNN for workpiece surface defect recognition, with the synaptic weights modeled using the experimentally characterized multilevel resistance states (Figure 5a). In this simulation framework, the measured 128 distinct nonvolatile conductance levels (7 bits) serve as the discrete weight quantization levels in the CNN, while the experimentally obtained linearity and symmetry of the resistance modulation are incorporated into the weight update rules. The CNN architecture, consisting of two convolutional layers for feature extraction and two fully connected layers for classification, was trained on a labeled dataset of workpiece defect images via a backpropagation algorithm. Defects are classified into the following six categories: crazing, inclusion, patches, pitted surface, rolled-in scale, and scratches (labeled a–f, respectively, in the confusion matrix). During the inference simulation, the forward propagation was emulated by mapping the trained weight matrices onto the simulated conductance matrix, where multiply accumulate operations were computed according to Kirchhoff’s current law to emulate in-memory analog computing. The simulation results demonstrate that the device’s multilevel capability enables the CNN to achieve a high classification accuracy of 97.1% after only 40 training epochs (Figure 5b) in detecting micro-defects on workpiece surfaces. Moreover, the trained weights were quantized to 6-bit for the six defect categories. Upon inference testing with 100 test images, the inference accuracy exceeded 90% for each defect category, as illustrated in the confusion matrix in Figure 5c. These results confirm the effectiveness of the FeS-FET array in recognizing micro-defects on workpiece surfaces. Furthermore, by incorporating the measured ultrafast switching speed (20 ns), this simulation study validates, at the system level, that the Te/Fe_3_GaTe_2_ FeS-FET platform holds great promise for high-speed, high-precision industrial defect inspection.

## 3. Conclusions

In summary, we have demonstrated a ferroelectric semiconductor field-effect transistor based on a 1D-2D lateral Te/Fe_3_GaTe_2_ heterojunction. The Te nanowire retains robust ferroelectricity after heterojunction integration, as confirmed by piezoresponse force microscopy. Driven by the vertical remanent polarization of Te, which modulates the in-plane resistance of the channel heterojunction, the device achieves continuously tunable nonvolatile resistance states exceeding 7 bits, an on/off ratio of 10^3^, long-term retention over 10^3^ s, ultrafast switching down to 20 ns and an ultrahigh information density of 1.19 Tbit in^−2^.Compared with other state-of-the-art ferroelectric and neuromorphic devices [17,18,19,31,46], our device exhibits highly competitive advantages in ultrafast switching and multi-state capacity, as summarized in Table S1. Furthermore, simulations based on the FeS-FET array achieve 97.1% accuracy in the identification of six types of micro-defects on workpiece surfaces. These results establish the Te/Fe_3_GaTe_2_ heterojunction as a promising platform for next-generation high-density nonvolatile memory and ultrafast neuromorphic computing.

## Data Availability

The original contributions presented in the study are included in the article and Supplementary Materials. Further inquiries and raw data supporting the reported results can be directed to the corresponding authors upon reasonable request.

## Supplementary Materials

The following supporting information can be downloaded at https://www.mdpi.com/article/10.3390/nano16150935/s1, Table S1: Performance benchmarking of our Te/Fe_3_GaTe_2_ 1D-2D FeS-FET against other state-of-the-art ferroelectric and neuromorphic devices; Figure S1: Ferroelectric hysteresis of Te nanowire on the heterojunction overlap; Figure S2: Statistical and noise analysis of the 128 resistance states and probability density distributions of several representative states. Figure S3: Nonvolatile memory retention characteristics of the FeS-FET.
