# Supplementary material for: Te/Fe3GaTe2 1D-2D Ferroelectric Heterojunction Transistors Enabling Ultrafast Multi-State Switching for Workpiece Surface Defect Inspection
Source: Nanomaterials (Basel). 2026 Jul 29;16(15):935. doi: 10.3390/nano16150935 (PMC13467920; doi:10.3390/nano16150935)
Supplement: Supplementary file 1 [file nanomaterials-16-00935-s001.zip › nanomaterials-4441227-supplementary.pdf]

Te/Fe_3GaTe_2 1D–2D ferroelectric heterojunction transistors enabling ultrafast multi-state switching for workpiece surface defect inspection

Shiqiang Wang [1,2,†], Zewei He [2,†], Tianyun Wang [2,†], ZiYu Gao [2], Lin Wang [1,*], Jinlei Zhang [2,*] and Yucheng Jiang [2]

Supplementary Text

Experimental Section

Synthesis of Te Nanowires

Single-crystal Tellurium (Te) nanowires were synthesized via a bottom-up hydrothermal method. Typically, sodium tellurite (Na_2TeO_3) was dissolved in deionized water under vigorous stirring, followed by the addition of polyvinylpyrrolidone (PVP) as a surfactant and hydrazine hydrate as a reducing agent. The mixture was transferred to a Teflon-lined stainless steel autoclave and heated at 180°C for 4 hours. The resulting product was washed repeatedly with deionized water and ethanol, and then dispersed in isopropyl alcohol for device fabrication.

Device Fabrication

The synthesized Te nanowires were drop-casted onto a highly doped silicon wafer covered with a 300 nm thermal oxide layer (SiO_2/Si). 2D metallic ferromagnet Fe_3GaTe_2 flakes were mechanically exfoliated from bulk crystals and precisely aligned and transferred onto one end of a targeted Te nanowire using a dry transfer platform with a PDMS stamp. Electrodes (Pt/Au, 10/60 nm) were patterned using ultraviolet (UV) photolithography followed by metal deposition and lift-off processes.

Characterization and Measurements

Raman spectra were recorded using a confocal Raman spectrometer with a 532 nm excitation laser. Piezoresponse force microscopy (PFM) characterizations, including phase/amplitude mapping and hysteresis loops, were performed on an atomic force microscope (AFM) in vertical off-field mode using conducting Pt/Ir-coated tips. For electrical transport characterizations and multi-level pulse writing tests, the device chips were securely packaged using a wire bonder to connect the electrode pads to standard chip carriers. Subsequently, electrical measurements were carried out by coupling the bonded devices with multiple models of Keithley source meters (such as the Keithley 4200-SCS semiconductor parameter analyzer) within a vacuum probe station.

Table S1. Performance benchmarking of our Te/Fe_3GaTe_2 1D-2D FeS-FET against other state-of-the-art ferroelectric and neuromorphic devices.

Device Structure / Materials	Switching Speed	Number of States	Retention Time	Endurance	Application Demo	Ref.
α-In_2Se_3 FeS-FET (2D)	~ 40 ns	Analog (Continuous)	> 10^5 s	10^5 cycles	-	[31]
MoS_2 / $HfZrO_2$ FeFET (2D/3D)	~ 100 ns	32 states (5 bits)	> 10^4 s	10^4 cycles	Neuromorphic Computing	[46]
Oxide-based Synapse	~ 100 ns	16 states (4 bits)	> 10^4 s	-	Pattern Recognition	[17]
Phase-Change Memory (PCM)	~ 20 ns	Multiple states	> 10^5 s	10^6 cycles	Artificial Neural Network	[18]
Halide Perovskite Synapse	~ 1 μs	8 states (3 bits)	> 10^3 s	10^3 cycles	Image Processing	[19]
**Te / Fe_3GaTe_2 (1D/2D)**	**20 ns**	**128 states (>7 bits)**	**> 3000 s**	**> 100 cycles**	**Defect Inspection (97.1%)**	**This work**

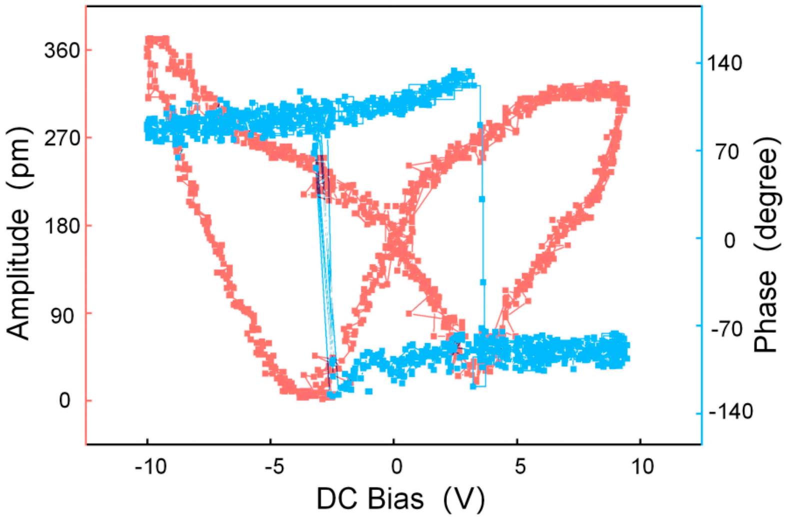

Figure S1: Ferroelectric hysteresis of Te nanowire on the heterojunction overlap.

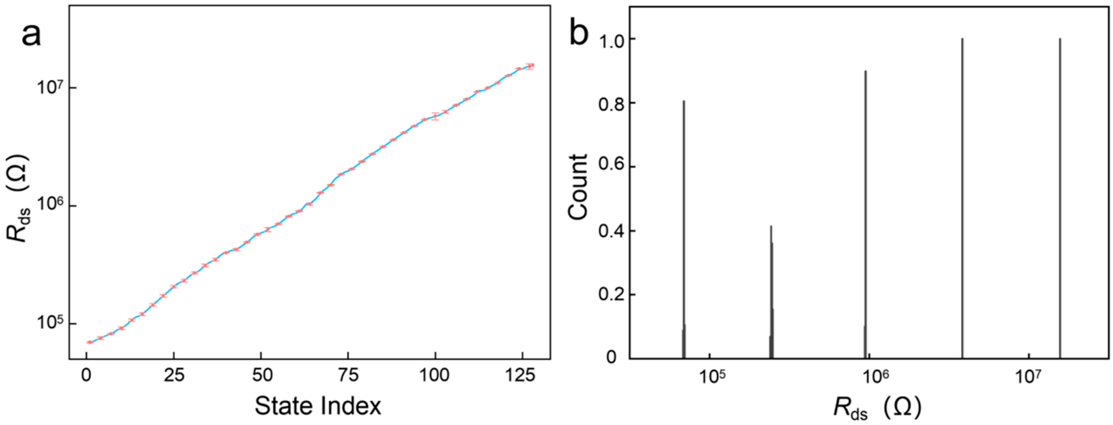

Figure S2: Statistical and noise analysis of the 128 resistance states and probability density distributions of several representative states. (a) Mean resistance values of the 128 states extracted from the retention measurements. The error bars represent ± 3σ standard deviations, indicating no statistical overlap between adjacent states. (b) Probability density distributions of several representative states (e.g., states 1, 25, 50, 75, 100, and 128), further confirming the distinct separation and low read noise of the multi-level switching behavior.

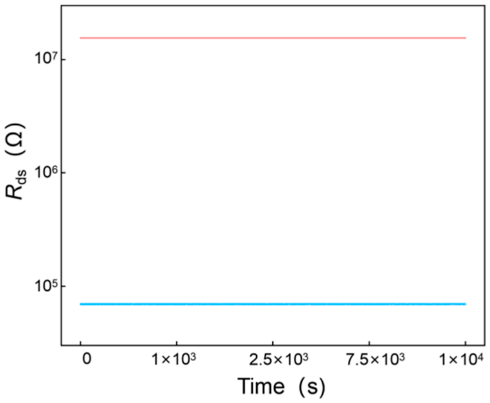

Figure S3: Long-term retention of HRS/LRS in the FeS-FET. Long-term retention of the channel resistance R_{ds} in HRS and LRS measured over 10^4 s at room temperature, demonstrating excellent nonvolatile stability with a distinct memory window.

References

17. Wang, Z.; Joshi, S.; Savel'ev, S.E.; Jiang, H.; Midya, R.; Lin, P.; Hu, M.; Ge, N.; Strachan, J.P.; Li, Z.; et al. Memristors with Diffusive Dynamics as Synaptic Emulators for Neuromorphic Computing. *Nat. Mater*. **2017**, 16, 101–108.

18. Sebastian, A.; Le Gallo, M.; Khaddam-Aljameh, R.; Eleftheriou, E. Memory Devices and Applications for In-Memory Computing. *Nat. Nanotechnol*. **2020**, 15, 529–544.

19. Bisquert, J.; Guerrero, A. Dynamic Instability and Time Domain Response of a Model Halide Perovskite Memristor for Artificial Neurons. J. *Phys. Chem. Lett*. **2022**, 13, 3789–3795.

31. Si, M.; Saha, A.K.; Gao, S.; Qiu, G.; Qin, J.; Duan, Y.; Jian, J.; Niu, C.; Wang, H.; Wu, W.; et al. A Ferroelectric Semiconductor Field-Effect Transistor. Nat. *Electron*. **2019**, 2, 580–586.

46. Huang, X.; Tong, L.; Xu, L.; Shi, W.; Peng, Z.; Li, Z.; Yu, X.; Li, W.; Wang, Y.; Zhang, X.; et al. 2D MoS_2-Based Reconfigurable Analog Hardware. *Nat. Commun*. **2025**, 16, 101.
